# Relation of microvascular dysfunction to exercise capacity and symptoms in patients with severe aortic stenosis

**DOI:** 10.1186/1532-429X-13-S1-O5

**Published:** 2011-02-02

**Authors:** Christopher D Steadman, Michael Jerosch-Herold, Benjamin Grundy, Suzanne Rafelt, Leong L Ng, Iain B Squire, Nilesh J Samani, Gerry P McCann

**Affiliations:** 1 Department of Cardiovascular Sciences, University of Leicester, Leicester, UK; 2 Brigham and Women's Hospital and Harvard Medical School, Boston, MA, USA; 3 NIHR Leicester Cardiovascular Biomedical Research Unit, Leicester, UK

## Objective

The aim of this study was to assess the impact of left ventricular hypertrophy, myocardial fibrosis, myocardial perfusion reserve (MPR) and diastolic dysfunction on objectively measured aerobic exercise capacity (peak VO_2_) in severe aortic stenosis (AS).

## Background

The management of asymptomatic patients with severe AS remains controversial and clinical practice varies. Echocardiographic measures of severity do not discriminate between symptomatic status or predict exercise capacity. The purpose of this study was to investigate the mechanisms contributing to symptom generation and exercise intolerance. This needs to be fully understood to optimise the management of asymptomatic AS.

## Methods

Patients were prospectively enrolled from a single cardiac surgical centre. Inclusion criteria: age 18-85, isolated severe AS referred for valve replacement. Exclusion criteria: syncope; other moderate/severe valve disease, previous valve surgery, obstructive coronary artery disease (>50% luminal stenosis on invasive angiography), chronic obstructive pulmonary disease, atrial fibrillation, estimated glomerular filtration rate <30mL/min. Investigations and primary outcome measures; cardiac magnetic resonance (CMR) - left ventricular mass index (LVMI), MPR (calculated from absolute myocardial blood flow during adenosine hyperaemia and rest determined by model-independent deconvolution of signal intensity curves with an arterial input function), late gadolinium enhancement (LGE); echocardiography - AS severity, tissue Doppler-derived diastolic function; symptom-limited bicycle ergometer cardiopulmonary exercise testing (CPEX) - peak VO_2_. Linear regression investigated possible predictors of continuous outcome measures. Stepwise selection methods were used to determine the most important predictors of outcome.

## Results

**Figure 1:**
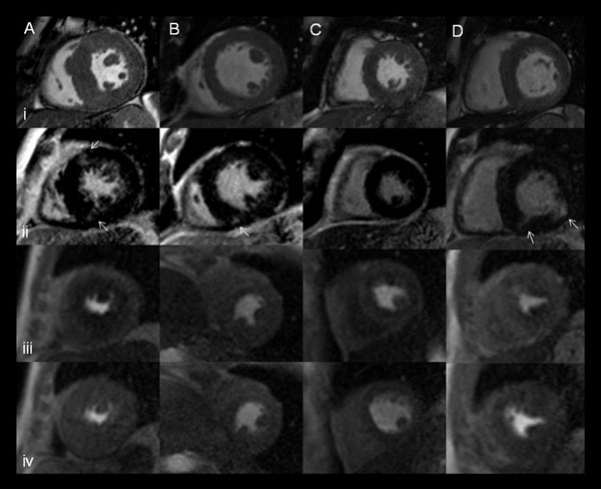
Patients (A-D). i) Short-axis cine end-diastole; ii) LGE - white arrows; Perfusion imaging mid-LV slice iii) hyperaemia, iv) rest.

**Figure 2:**
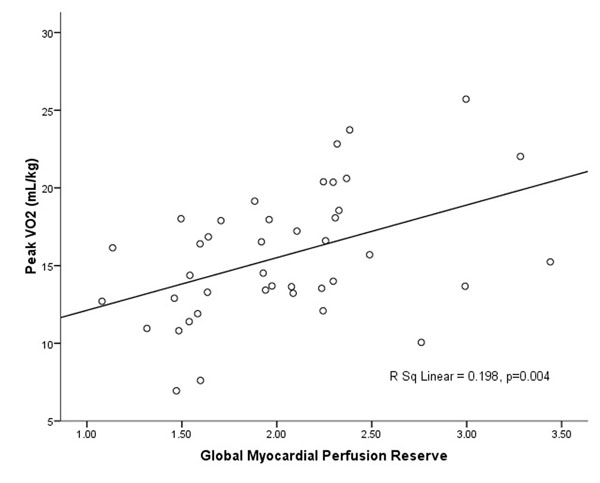
Peak VO_2_ and MPR

Four patients with variable LVMI, LGE and MPR are shown, Figure 1. Univariate analyses and results from the stepwise model selection for peak VO_2_ are summarised in Table 1. Only MPR was of independent significance in predicting age and sex corrected peak VO_2_. The relationship between peak VO_2_ and MPR is shown, Figure 2. Patients with higher NYHA Class had lower MPR (p=0.001). Examining predictors of MPR the best stepwise model contained LVMI and LGE category as independent predictors, Table 2.

**Table 1:** Predictors of Peak VO2

Variable	Univariate β	p-value	Stepwise model β	p-value
Sex	-0.41	0.005	-0.436	0.002
Age	-0.32	0.03	-0.154	0.248
Peak aortic velocity	-0.18	0.24	na	na
Aortic valve area index	0.04	0.79	na	na
Septal E/E’	-0.35	0.01	na	na
LV mass index	0.03	0.85	na	na
LV ejection fraction	-0.02	0.91	na	na
Late gadolinium enhancement	-0.023	0.517	na	na
Myocardial perfusion reserve	0.45	0.004	0.457	0.001

**Table 2:** Predictors of Perfusion Reserve

Variable	Univariate β	p-value	Stepwise model β	p-value
Sex	0.38	0.023	na	na
Age	-0.093	0.538	na	na
Diastolic perfusion time	-0.399	0.01	na	na
Peak aortic velocity	-0.339	0.02	na	na
Aortic valve area index	0.209	0.172	na	na
LV mass index	-0.516	<0.001	-0.403	0.004
LV ejection fraction	0.259	0.086	na	na
Late gadolinium enhancement	-0.456	0.002	-0.306	0.025
Septal E/E’	-0.312	0.041	na	na

## Conclusions

MPR is a novel independent predictor of peak VO_2_ and is inversely related to NYHA functional class in severe AS. Microvascular dysfunction is determined by a combination of factors including AS severity, LVMI, diastolic perfusion time, myocardial fibrosis and LV filling pressure. Further work is required to determine the clinical significance of microvascular dysfunction in AS.

